# Accuracy of Ultrasound in Estimating Uterine Fibroid Size: A Correlative Study With Intraoperative Findings

**DOI:** 10.7759/cureus.115376

**Published:** 2026-08-28

**Authors:** Viral B Makwana, Prafull D Patil, Abhay S Chandibhamar

**Affiliations:** 1 Obstetrics and Gynecology, Smt. B.K. Shah Medical Institute &amp; Research Centre, Sumandeep Vidyapeeth (Deemed to be University), Vadodara, IND; 2 Radiology, Smt. B.K. Shah Medical Institute &amp; Research Centre, Sumandeep Vidyapeeth (Deemed to be University), Vadodara, IND; 3 Radiology, Dhiraj Hospital, Smt. B.K. Shah Medical Institute &amp; Research Centre, Sumandeep Vidyapeeth (Deemed to be University), Vadodara, IND

**Keywords:** diagnostic accuracy, diagnostic imaging, intraoperative findings, pelvic ultrasonography, preoperative planning, uterine leiomyoma

## Abstract

Background

Uterine fibroids are the most common benign tumors of the female reproductive tract and frequently require surgical management. Accurate preoperative estimation of fibroid size is essential for selecting the optimal surgical approach, anticipating operative complexity, and improving surgical outcomes. Ultrasonography remains the most widely used first-line imaging modality because of its accessibility, cost-effectiveness, and noninvasive nature. However, its ability to accurately reflect true intraoperative fibroid size may vary.

Objective

To evaluate the accuracy of preoperative ultrasonography in estimating uterine fibroid size by comparing ultrasonographic measurements with intraoperative findings.

Materials and methods

This prospective observational correlational study included women diagnosed with uterine fibroids who were scheduled for elective myomectomy or hysterectomy at a tertiary care center. The maximum diameter of the dominant fibroid measured on preoperative ultrasonography was compared with the corresponding intraoperative measurement, which served as the reference standard. Statistical analysis included the paired t-test, Pearson's correlation coefficient, intraclass correlation coefficient (ICC), Bland-Altman analysis, and subgroup analysis according to fibroid size and type.

Results

Eighty participants with complete paired measurements were included in the analysis. The mean ultrasonographic fibroid diameter was 5.20 ± 2.10 cm, compared with an intraoperative diameter of 5.52 ± 2.18 cm. Ultrasonography underestimated fibroid size by a mean of 0.32 ± 0.88 cm. This difference was statistically significant (t(79) = −3.25, p = 0.0017), with a 95% confidence interval (CI) ranging from −0.52 to −0.12 cm. Ultrasonographic and intraoperative measurements demonstrated strong positive correlation and excellent agreement. Overall, 62 (77.5%) participants had ultrasonographic measurements within ±1 cm of the intraoperative measurement. Greater measurement discrepancies were observed among intramural fibroids.

Conclusion

Preoperative ultrasonography demonstrated strong correlation and excellent agreement with intraoperative measurements of uterine fibroid size. Although it showed a small but statistically significant tendency to underestimate fibroid size, particularly in intramural fibroids, ultrasonography remains a reliable first-line imaging modality for routine preoperative assessment. Awareness of its limitations may facilitate more accurate surgical planning, and supplementary imaging may be considered in selected complex cases.

## Introduction

Uterine fibroids, also known as leiomyomas, are the most common benign tumors of the female reproductive tract and affect a substantial proportion of women during their reproductive years. These tumors arise from the smooth muscle cells of the myometrium and vary considerably in size, number, and anatomical location within the uterus. Although many fibroids remain asymptomatic, a significant proportion of women present with abnormal uterine bleeding, pelvic pain, pressure symptoms, infertility, and adverse pregnancy outcomes, frequently necessitating surgical intervention, particularly myomectomy in women wishing to preserve fertility [1].

Accurate preoperative assessment of fibroid size is essential for optimal clinical management because fibroid size influences symptom severity, selection of the surgical approach, estimation of operative difficulty, anticipated intraoperative blood loss, and the risk of recurrence [2]. Consequently, imaging plays a pivotal role in treatment planning. Ultrasonography, performed using transabdominal and/or transvaginal approaches, remains the preferred first-line imaging modality because it is widely available, noninvasive, cost-effective, and free from ionizing radiation [3].

Despite these advantages, ultrasonography has inherent limitations that may affect measurement accuracy. Factors such as operator dependency, obesity, uterine position, coexisting adenomyosis, multiple fibroids, and deeply located intramural lesions may reduce the accuracy of fibroid detection and size estimation. Small fibroids or those embedded within the myometrium may be underestimated or occasionally missed during routine ultrasonographic examination [4]. Magnetic resonance imaging (MRI) provides superior soft-tissue contrast and more comprehensive fibroid mapping; however, its routine use is restricted by higher cost, limited availability, and longer acquisition times, particularly in resource-limited healthcare settings [5]. Nevertheless, recent evidence continues to support ultrasonography as the preferred first-line imaging modality because of its accessibility and cost-effectiveness despite the superior anatomical detail offered by MRI [6].

Direct intraoperative assessment during myomectomy permits visualization and measurement of fibroids and is therefore considered the reference standard for evaluating fibroid size. Previous studies have demonstrated discrepancies between preoperative imaging findings and intraoperative observations, particularly in patients with multiple fibroids and in minimally invasive procedures where tactile feedback is reduced [7]. Residual fibroids left at surgery may subsequently enlarge, leading to recurrence of symptoms and the need for repeat intervention [8].

Given the widespread reliance on ultrasonography for preoperative evaluation, it is important to determine how accurately ultrasonographic measurements reflect true intraoperative fibroid size. Demonstrating close agreement would strengthen confidence in ultrasonography as the primary imaging modality, whereas identifying systematic discrepancies could support the selective use of supplementary imaging and facilitate improved surgical planning. Therefore, the present study aimed to evaluate the accuracy of preoperative ultrasonography in estimating uterine fibroid size by correlating ultrasonographic measurements with intraoperative findings.

We hypothesized that preoperative ultrasonography would demonstrate strong correlation and agreement with intraoperative fibroid measurements while exhibiting a small tendency to underestimate fibroid size. We also explored whether measurement accuracy differed according to fibroid size and fibroid type.

Evidence regarding the accuracy of ultrasonography for fibroid size estimation from Indian tertiary care settings, especially from Gujarat, remains limited. Furthermore, few studies have evaluated measurement accuracy according to fibroid size and type within this population. The present study therefore sought to provide clinically relevant evidence that may improve preoperative planning and intraoperative decision-making in resource-limited settings.

## Materials and methods

Study design and setting

This prospective observational correlational study was conducted in the Department of Radiology, Dhiraj Hospital, Vadodara, Gujarat, India, between May 2026 and July 2026. Ethical approval was obtained from the Institutional Ethics Committee (IEC) before commencement of participant recruitment and data collection (approval no.: SVIEC/ON/Medi/RP/May/26/64).

Study population

The study population comprised women aged 18 years or older who were diagnosed with uterine fibroids on preoperative ultrasonography and were scheduled to undergo elective myomectomy or hysterectomy at Dhiraj Hospital during the study period. Consecutive eligible participants who fulfilled the inclusion criteria and provided written informed consent were enrolled.

Patients with suspected or confirmed uterine malignancy, pregnancy, previous uterine surgery that could significantly alter pelvic anatomy, or incomplete intraoperative fibroid measurements were excluded. All enrolled participants underwent standardized preoperative ultrasonography followed by surgical management. The dominant fibroid identified on ultrasonography was matched with the corresponding intraoperative fibroid and included in the paired analysis.

Inclusion criteria

The following are the inclusion criteria: women aged 18 years or older; ultrasonographic diagnosis of one or more uterine fibroids; scheduled for elective myomectomy or hysterectomy; and willingness to participate in the study and provision of written informed consent.

Exclusion criteria

The following are the exclusion criteria: suspected or confirmed uterine malignancy; pregnancy; previous uterine surgery likely to alter uterine anatomy and interfere with accurate fibroid measurement; incomplete intraoperative assessment or inability to obtain reliable intraoperative measurements of the dominant fibroid.

Sample size estimation

The sample size was calculated for the primary outcome, namely, the mean paired difference between preoperative ultrasonographic and intraoperative measurements of the dominant uterine fibroid. The calculation was performed using the standard formula for comparing paired means with a two-sided paired t-test, as described by Chow et al. [9].

Here,

• n = required number of paired observations

• Zα/2 = standard normal deviate corresponding to a two-sided significance level of 5% (1.96)

• Zβ = standard normal deviate corresponding to 80% statistical power (0.84)

• σd = anticipated standard deviation of the paired differences

• Δ = minimum clinically important mean difference

Substituting the assumed values:

*n = [(1.96 + 0.84) &times; 1.5 / 0.5]&sup2;*

*= (2.80 &times; 1.5 / 0.5)&sup2;*

*= (8.40)&sup2;*

*= 70.56*

Accordingly, the minimum required sample size was 71 paired observations. To compensate for an anticipated 10% attrition rate, the required recruitment target was calculated as 71/(1 − 0.10) = 78.9. Therefore, at least 79 participants were required, and the target sample size was rounded up to 80 participants to account for possible exclusions.

For the sample-size calculation, an anticipated standard deviation of the paired differences of 1.5 cm was assumed. A mean paired difference of 0.5 cm was selected a priori as the minimum difference of interest for sample-size estimation. These values were used as study-specific planning assumptions rather than as thresholds directly established by Levens et al. [1] and Battista et al. [4].

Ethical considerations

The study protocol was reviewed and approved by the Institutional Ethics Committee (IEC) of Dhiraj Hospital, Vadodara, Gujarat, India (approval no.: SVIEC/ON/Medi/RP/May/26/64). Written informed consent was obtained from all participants after explaining the study objectives, procedures, potential benefits, and possible risks. Participant confidentiality was maintained by anonymizing all collected data. The study was conducted in accordance with the ethical principles of the Declaration of Helsinki and its subsequent amendments.

Preoperative ultrasonographic assessment

All participants underwent standardized preoperative ultrasonographic evaluation using transabdominal and/or transvaginal approaches according to uterine size, fibroid characteristics, and patient-related factors. The preoperative ultrasonographic examination was generally performed approximately one to two days before surgery. All examinations and measurements were performed using the same ultrasound system by a single experienced radiologist following a standardized imaging protocol, thereby minimizing interobserver variability. During each examination, the number, anatomical location (anterior, posterior, fundal, lateral/cornual, or cervical), type (submucosal, intramural, or subserosal), and maximum diameter of each identified fibroid were systematically assessed and recorded on the structured study proforma. In participants with multiple fibroids, the largest fibroid was predefined as the dominant index fibroid for comparison with the corresponding intraoperative measurement.

Although all fibroids were documented during ultrasonographic examination, only the dominant fibroid from each participant was included in the statistical analysis to ensure one independent pair of observations per participant.

Surgical procedure and intraoperative assessment

All surgical procedures were performed by the same gynecologic surgeons, who performed and recorded the intraoperative fibroid measurements for all study participants, according to standard institutional protocols. During surgery, the uterus was systematically inspected and palpated to identify visible and palpable fibroids. Following enucleation or removal, the maximum diameter of each identified fibroid was measured immediately using a sterile measuring scale and recorded on the structured study proforma. The dominant fibroid identified preoperatively was matched with the corresponding intraoperative fibroid based on anatomical location, fibroid type, and relative size. The maximum intraoperative diameter of the matched dominant fibroid served as the reference standard for comparison with the corresponding preoperative ultrasonographic measurement.

Data collection

Data were recorded using a predesigned structured data collection proforma (Appendix 1). Information collected included demographic characteristics, clinical presentation, body mass index (BMI), parity, ultrasonographic findings (number, location, type, and maximum diameter of fibroids), operative details, and corresponding intraoperative measurements. Where multiple fibroids were present, all lesions were documented; however, only the dominant fibroid was included in the paired statistical analysis. This approach ensured statistical independence of observations and prevented clustering arising from multiple fibroids within the same participant.

Outcome measures

Primary Outcome

The primary outcome was the mean paired difference between preoperative ultrasonographic and intraoperative measurements of the maximum diameter of the dominant uterine fibroid. Agreement between the two measurement methods, assessed using the intraclass correlation coefficient (ICC) and Bland-Altman analysis, was evaluated as a secondary outcome.

Secondary Outcomes

The secondary outcomes included the following: Pearson's correlation between ultrasonographic and intraoperative measurements; ICC for absolute agreement; Bland-Altman mean bias and 95% limits of agreement (LoA); absolute measurement error; proportion of measurements within predefined accuracy thresholds (±0.5 cm, ±1.0 cm, and ±2.0 cm); and comparison of measurement error according to fibroid size and fibroid type.

Statistical analysis

Data were entered into Microsoft Excel (Microsoft Corporation, Redmond, WA) and analyzed using Statistical Product and Service Solutions (SPSS, version 27.0; IBM SPSS Statistics for Windows, Armonk, NY).

Continuous variables were summarized as mean ± standard deviation (SD), whereas categorical variables were expressed as number (percentage), n (%). The paired difference was calculated as follows: Ultrasonographic measurement − Intraoperative measurement

Normality of the paired differences was assessed using the Shapiro-Wilk test together with visual inspection of histograms and normal probability (Q-Q) plots before applying parametric analysis.

The mean paired difference between ultrasonographic and intraoperative measurements was evaluated using the paired t-test. The strength of the linear relationship between the two measurement methods was assessed using Pearson's correlation coefficient (r). Agreement between ultrasonographic and intraoperative measurements was further evaluated using the ICC based on an absolute-agreement two-way mixed-effects model, together with Bland-Altman analysis, which was used to estimate the mean measurement bias and the 95% LoA.

Absolute measurement error was calculated as the absolute value of the paired difference between ultrasonographic and intraoperative measurements. Differences in mean absolute error among fibroid size categories and fibroid types were evaluated using one-way analysis of variance (ANOVA). When the overall comparison was statistically significant, appropriate post-hoc pairwise comparisons were performed. All statistical tests were two-sided, and a p-value of <0.05 was considered statistically significant.

## Results

Participant flow

A total of 86 women were assessed for eligibility during the study period. Of these, 80 participants fulfilled the eligibility criteria, provided written informed consent, and completed both preoperative ultrasonography and surgery with complete intraoperative measurements. Six participants were excluded because of incomplete intraoperative measurements (n = 3), withdrawal of consent (n = 2), or conversion to emergency surgery (n = 1). In participants with multiple fibroids, only the dominant (largest) fibroid was included in the paired analysis, resulting in 80 paired ultrasonographic and intraoperative measurements.

Baseline characteristics

The baseline demographic and clinical characteristics of the study participants are summarized in Table 1. The mean age of the participants was 38.6 ± 6.9 years, and the mean body mass index (BMI) was 25.8 ± 3.7 kg/m². Heavy menstrual bleeding was the most common presenting complaint, reported by 52 (65.0%) participants, followed by pelvic pain in 38 (47.5%), pressure or bulk symptoms in 34 (42.5%), and infertility or subfertility in 18 (22.5%) participants. The mean number of fibroids per participant was 2.8 ± 1.6, while 50 (62.5%) participants had multiple fibroids. Myomectomy was performed in 46 (57.5%) participants, whereas 34 (42.5%) underwent hysterectomy.

**Table 1:** Baseline demographic and clinical characteristics of the participants (n = 80) Participants could report more than one presenting symptom. SD = Standard deviation

Variable	Value
Age (years), mean ± SD	38.6 ± 6.9
Body mass index (kg/m²), mean ± SD	25.8 ± 3.7
Parity, n (%)	
Nulliparous	22 (27.5%)
Multiparous	58 (72.5%)
Presenting symptoms, n (%)*	
Heavy menstrual bleeding	52 (65.0%)
Pelvic pain	38 (47.5%)
Pressure/bulk symptoms	34 (42.5%)
Infertility/subfertility	18 (22.5%)
Surgical procedure, n (%)	
Myomectomy	46 (57.5%)
Hysterectomy	34 (42.5%)
Number of fibroids per participant, mean ± SD	2.8 ± 1.6
Multiple fibroids	50 (62.5%)

Ultrasonographic characteristics of the dominant fibroid

The ultrasonographic characteristics of the dominant fibroid are presented in Table 2. Intramural fibroids constituted the largest subgroup, occurring in 46 (57.5%) participants, followed by subserosal fibroids in 20 (25.0%) and submucosal fibroids in 14 (17.5%) participants. With respect to anatomical location, posterior wall fibroids were the most common (28, 35.0%), followed by anterior wall fibroids (26, 32.5%). The largest fibroid measured >6 cm in 28 (35.0%) participants.

**Table 2:** Ultrasonographic characteristics of the dominant fibroid (n = 80) USG = Ultrasonography

Characteristic	n (%)
Anatomical location	
Anterior wall	26 (32.5%)
Posterior wall	28 (35.0%)
Fundal	18 (22.5%)
Lateral/cornual	8 (10.0%)
Fibroid type	
Submucosal	14 (17.5%)
Intramural	46 (57.5%)
Subserosal	20 (25.0%)
Largest fibroid size (USG)	
<4 cm	24 (30.0%)
4-6 cm	28 (35.0%)
>6 cm	28 (35.0%)

The comparison between preoperative ultrasonographic and intraoperative measurements of the dominant fibroid is presented in Table 3. The mean maximum fibroid diameter measured by ultrasonography was 5.20 ± 2.10 cm, whereas the corresponding intraoperative mean diameter was 5.52 ± 2.18 cm. The mean paired difference (ultrasonography minus intraoperative measurement) was −0.32 ± 0.88 cm, indicating a small tendency for ultrasonography to underestimate fibroid size.

**Table 3:** Comparison of ultrasonographic and intraoperative measurements of the dominant fibroid (n = 80) CI = Confidence interval; df = Degrees of freedom; SD = Standard deviation

Parameter	Ultrasonographic Mean ± SD	Intraoperative Mean ± SD	Mean Paired Difference (USG − Intraoperative)	t	df	Exact p value	95% CI
Maximum diameter (cm)	5.20 ± 2.10	5.52 ± 2.18	−0.32 ± 0.88	−3.25	79	0.0017	−0.52 to −0.12

Normality assessment of the paired differences using the Shapiro-Wilk test demonstrated a statistically significant departure from normality (W = 0.961, p = 0.0145). Visual inspection of the histogram and normal Q-Q plot showed only mild deviation from normality. Given the sample size (n = 80), the paired t-test was considered appropriate because of its robustness to modest departures from normality. The paired t-test demonstrated a statistically significant difference between ultrasonographic and intraoperative measurements (mean difference = −0.32 cm, 95% CI: −0.52 to −0.12 cm; t(79) = −3.25, p = 0.0017).

The agreement between ultrasonographic and intraoperative measurements is summarized in Table 4. Ultrasonographic measurements demonstrated a strong positive correlation with intraoperative measurements (Pearson's correlation coefficient, r = 0.93, p < 0.001). Agreement between the two methods was excellent, with an ICC of 0.91 (95% CI: 0.86-0.94; p < 0.001).

**Table 4:** Correlation and agreement between ultrasonographic and intraoperative measurements

Statistic	Estimate	95% CI	Exact p value
Pearson's correlation coefficient (r)	0.93	—	<0.001
Coefficient of determination (r²)	0.86	—	—
Intraclass correlation coefficient (ICC)	0.91	0.86–0.94	<0.001
Bland-Altman mean bias (cm)	−0.32	−0.52 to −0.12	—
95% limits of agreement (cm)	−2.05 to +1.41	—	—

Bland-Altman analysis (Figure 1) demonstrated a mean bias of −0.32 cm, with 95% limits of agreement ranging from −2.05 cm to +1.41 cm, indicating that individual ultrasonographic measurements could range from approximately 2.05 cm below to 1.41 cm above the corresponding intraoperative measurement.

**Figure 1:**
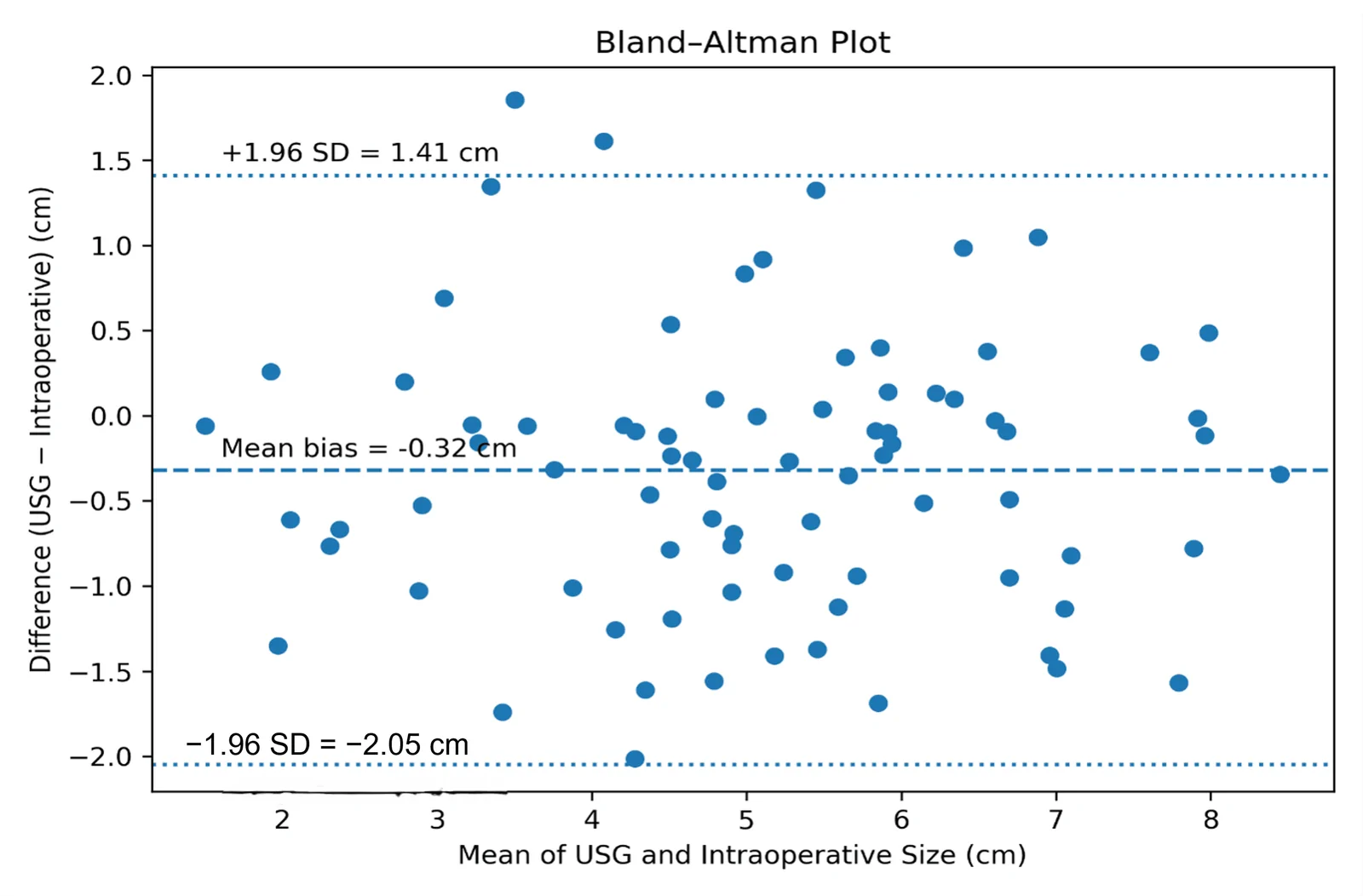
Bland-Altman plot demonstrating agreement between ultrasonographic and intraoperative measurements of the dominant uterine fibroid The central dashed line represents the mean bias (−0.32 cm), while the upper and lower dashed lines indicate the 95% limits of agreement (−2.05 cm to +1.41 cm).

Excellent agreement was observed between ultrasonographic and intraoperative measurements, with an ICC of 0.91 (95% CI: 0.86-0.94; p < 0.001).

The distribution of absolute measurement error is presented in Table 5. Ultrasonographic measurements were within ±0.5 cm of the intraoperative measurement in 42 (52.5%) participants, within ±1.0 cm in 62 (77.5%), and within ±2.0 cm in 76 (95.0%) participants. Only four (5.0%) participants demonstrated an absolute measurement difference greater than 2 cm.

**Table 5:** Absolute measurement error between ultrasonographic and intraoperative measurements (n = 80) Categories are cumulative and should not be summed.

Accuracy threshold	n (%)
Within ±0.5 cm	42 (52.5%)
Within ±1.0 cm	62 (77.5%)
Within ±2.0 cm	76 (95.0%)
Greater than 2.0 cm	4 (5.0%)

The mean absolute measurement error according to intraoperative fibroid size and fibroid type is presented in Table 6. There was no statistically significant difference in mean absolute measurement error across fibroid size categories (F(2,77) = 0.131, p = 0.877). In contrast, the mean absolute measurement error differed significantly according to fibroid type (F(2,77) = 34.854, p < 0.001, η² = 0.475). Tukey's post-hoc analysis demonstrated significantly greater measurement error for intramural fibroids than for submucosal fibroids (adjusted p < 0.001) and subserosal fibroids (adjusted p < 0.001), whereas no significant difference was observed between submucosal and subserosal fibroids (adjusted p = 0.857).

**Table 6:** Mean absolute measurement error according to intraoperative fibroid size and fibroid type (n = 80) Values are expressed as mean ± standard deviation (SD). Fibroid size categories in this analysis were based on the intraoperative maximum diameter of the dominant fibroid.

Subgroup	n	Mean Absolute Error (cm), Mean ± SD	Test statistic	df	Exact p value
Fibroid size					
<4 cm	26	0.75 ± 0.64			
4-6 cm	24	0.66 ± 0.62			
>6 cm	30	0.71 ± 0.57	F = 0.131	2, 77	0.877
Fibroid type					
Submucosal	14	0.18 ± 0.06			
Intramural	46	1.07 ± 0.57			
Subserosal	20	0.26 ± 0.13	F = 34.854	2, 77	<0.001

Comparisons among groups were performed using one-way ANOVA. Significant ANOVA results were followed by Tukey's post-hoc pairwise comparisons. Fibroid size categories in this analysis were based on the intraoperative maximum diameter of the dominant fibroid.

## Discussion

Accurate preoperative estimation of uterine fibroid size is fundamental for selecting the optimal surgical approach, anticipating operative complexity, estimating intraoperative blood loss, and counselling patients regarding expected surgical outcomes. In the present study, preoperative ultrasonography demonstrated strong correlation and excellent agreement with intraoperative measurements of the dominant fibroid. Although ultrasonography showed a small but statistically significant tendency to underestimate fibroid size, 62 (77.5%) measurements fell within ±1 cm of the corresponding intraoperative measurement. These findings support the continued use of ultrasonography as the primary imaging modality for routine preoperative assessment.

The observed mean paired difference of −0.32 cm indicates that ultrasonography systematically underestimated fibroid size by a small margin. Although the mean difference is unlikely to influence clinical decision-making in most patients, individual measurement discrepancies may become clinically relevant when precise preoperative size estimation is required for surgical planning. Importantly, measurement error did not differ significantly across fibroid size categories in the present study. Similar measurement discrepancies have been reported previously and may be influenced by operator dependency, indistinct lesion margins, fibroid multiplicity, internal heterogeneity or degenerative change, and calcification with associated acoustic shadowing. These factors may impair visualization of fibroid boundaries and contribute to variability in ultrasonographic size estimation, particularly in anatomically complex or deeply situated lesions [10-12].

Our findings are consistent with previous studies demonstrating that ultrasonography is reliable for routine uterine fibroid assessment but may be less accurate in the presence of multiple fibroids or complex uterine anatomy [4,6,13]. Such discrepancies are clinically relevant because inaccurate preoperative estimation may affect expectations regarding fibroid size and operative planning. The present findings therefore emphasize the importance of recognizing circumstances in which ultrasonographic measurements should be interpreted with greater caution.

The Bland-Altman analysis performed in the present study provides additional insight beyond simple correlation analysis. Although the mean bias between ultrasonographic and intraoperative measurements was small, the 95% limits of agreement (−2.05 to +1.41 cm) indicate that individual ultrasonographic measurements may differ appreciably from the corresponding intraoperative measurements. This finding highlights an important distinction between correlation and agreement. Two methods may demonstrate excellent correlation while still exhibiting clinically relevant differences in individual measurements. Consequently, clinicians should consider both the average bias and the limits of agreement when interpreting preoperative ultrasonographic measurements [14,15].

Subgroup analysis demonstrated no statistically significant difference in measurement error across fibroid size categories, indicating that ultrasonographic accuracy was maintained over the range of fibroid sizes included in this study. In contrast, measurement error differed significantly according to fibroid type, with intramural fibroids exhibiting the greatest mean absolute error. The relatively higher error observed in intramural fibroids may be explained by their location within the myometrium, where lesion margins are often less clearly defined than those of submucosal or subserosal fibroids. Consequently, accurate delineation of intramural fibroids may be more challenging, particularly in enlarged uteri or in the presence of multiple fibroids [12]. These findings suggest that fibroid location and type, rather than fibroid size alone, may have a greater influence on ultrasonographic measurement accuracy.

From a clinical perspective, the findings of this study support the continued use of ultrasonography as the primary imaging modality for routine preoperative assessment of uterine fibroids. Its wide availability, noninvasive nature, absence of ionizing radiation, and relatively low cost make it particularly valuable in resource-limited healthcare settings. However, clinicians should recognize that ultrasonography may underestimate fibroid size in selected cases. Although measurement accuracy was not significantly influenced by fibroid size in the present study, intramural fibroids demonstrated greater measurement error than other fibroid types. In patients with complex uterine anatomy or when precise preoperative mapping is expected to influence surgical planning, supplementary magnetic resonance imaging (MRI) may provide additional anatomical detail [6,16].

The present study has several strengths. It employed a prospective design, used standardized ultrasonographic examinations performed by a single experienced radiologist, and compared measurements directly with intraoperative findings, which served as the reference standard. Restricting the primary analysis to the dominant fibroid ensured one paired observation per participant and avoided statistical clustering.

Nevertheless, certain limitations should be acknowledged. The study was conducted at a single tertiary care center, which may limit the generalizability of the findings. Only the dominant fibroid was included in the paired analysis, and MRI comparison was not performed because of resource constraints. In addition, although interobserver variability was minimized by using a single radiologist, the findings may not fully reflect routine clinical practice involving multiple operators. Future multicenter studies incorporating MRI comparison and assessment of interobserver variability would further strengthen the evidence regarding ultrasonographic accuracy in preoperative fibroid assessment [16,17]. Overall, the findings of the present study demonstrate that ultrasonography provides strong correlation and excellent agreement with intraoperative fibroid measurements, although individual measurement discrepancies were observed. Although a small degree of underestimation was observed, measurement accuracy remained consistent across different fibroid sizes. However, intramural fibroids exhibited significantly greater measurement error than submucosal and subserosal fibroids, highlighting the importance of careful interpretation in this subgroup. Ultrasonography therefore remains an appropriate first-line imaging modality for routine preoperative evaluation, with selective use of MRI reserved for anatomically complex cases or when detailed fibroid mapping is clinically indicated [18].

Limitations

This study has several limitations that should be considered while interpreting the findings. First, it was conducted at a single tertiary care center, which may limit the generalizability of the results to other clinical settings. Second, although ultrasonographic examinations were performed using a standardized protocol by a single experienced radiologist, the operator-dependent nature of ultrasonography limits assessment of interobserver variability. Third, only the dominant fibroid from each participant was included in the paired statistical analysis to maintain statistical independence; therefore, the findings may not fully reflect measurement accuracy in women with multiple smaller fibroids. Fourth, MRI was not performed for comparison because of resource limitations. Finally, intraoperative measurements obtained immediately after fibroid removal may have been influenced by minor tissue handling or deformation, although every effort was made to minimize measurement error. Detailed sonographic characteristics, such as echogenicity, internal heterogeneity or degenerative change, and calcification, were not systematically recorded as prespecified study variables. Therefore, their potential influence on visualization of fibroid margins and measurement discrepancy, including the effect of posterior acoustic shadowing associated with calcification, could not be specifically evaluated. Despite these limitations, the prospective design, standardized imaging protocol, and direct comparison with intraoperative measurements strengthen the validity of the study findings.

## Conclusions

Preoperative ultrasonography demonstrated strong correlation and excellent agreement with intraoperative measurements of dominant uterine fibroid size and remains a reliable first-line imaging modality for routine preoperative assessment. Although ultrasonography showed a small but statistically significant tendency to underestimate fibroid size, 62 (77.5%) measurements were within ±1 cm of the corresponding intraoperative measurement. Measurement accuracy did not differ significantly across fibroid size categories. However, intramural fibroids exhibited significantly greater measurement error than submucosal and subserosal fibroids, suggesting that fibroid type may influence ultrasonographic measurement accuracy. Clinicians should therefore interpret measurements of intramural fibroids with appropriate caution and consider supplementary MRI when detailed preoperative anatomical mapping is likely to influence surgical management. Recognition of potential measurement discrepancies may facilitate more informed preoperative surgical planning, patient counseling, and clinical decision-making.

Future multicenter studies incorporating larger sample sizes, assessment of interobserver variability, and direct MRI comparison are warranted to further validate these findings and optimize preoperative imaging strategies for women with uterine fibroids.

## Appendices

Appendix 1: Data collection proforma

Data Collected

Participant ID__________________________

Age (years)__________________________

Body Mass Index (kg/m²)__________________________

Parity☐ Nulliparous  ☐ Multiparous

Presenting Symptoms*☐ Heavy menstrual bleeding ☐ Pelvic pain ☐ Pressure/Bulk symptoms ☐ Infertility/Subfertility ☐ Other: __________

Number of Fibroids (USG)__________________________

Dominant Fibroid Location (USG)☐ Anterior ☐ Posterior ☐ Fundal ☐ Lateral/Cornual ☐ Cervical

Dominant Fibroid Type (USG)☐ Submucosal ☐ Intramural ☐ Subserosal

Maximum Diameter on USG (cm)__________________________

Planned Surgical Procedure☐ Myomectomy ☐ Hysterectomy

Dominant Fibroid Location (Intraoperative)__________________________

Dominant Fibroid Type (Intraoperative)__________________________

Maximum Intraoperative Diameter (cm)__________________________

Ultrasonographic Measurement (cm)__________________________

Intraoperative Measurement (cm)__________________________

Paired Difference (USG − Intraoperative) (cm)__________________________

Absolute Measurement Error (cm)__________________________

Additional Remarks__________________________________________________________

Abbreviations: USG = Ultrasonography.

* More than one presenting symptom could be be recorded for each participant.
